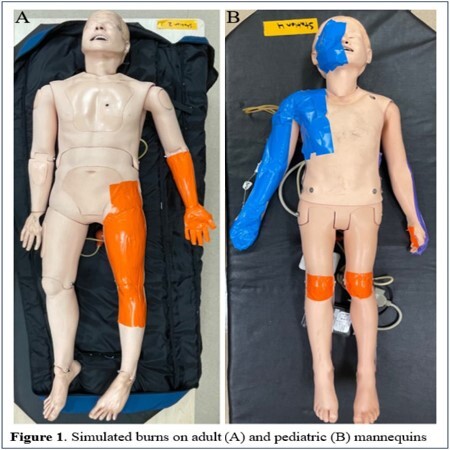# 536 A Novel Mobile Application for Burn Surface Area Calculation

**DOI:** 10.1093/jbcr/irae036.170

**Published:** 2024-04-17

**Authors:** Nicolas Malkoff, Brigette Cannata, Sarah Wang, Deborah Choe, Artur Manasyan, Lyle Koegler, Saman Kashani, Maxwell B Johnson, Haig A Yenikomshian, Justin Gillenwater

**Affiliations:** Keck School of Medicine of USC, Carlsbad, CA; Keck School of Medicine of USC, Staten Island, NY; Keck School of Medicine of USC, Los Angeles, CA; County of Los Angeles Fire Department, Mammoth Lakes, CA; Los Angeles County Fire Department, Monterey Park, CA; University of Southern California, Los Angeles, CA; Keck Medicine of USC, Los Angeles, CA; Keck School of Medicine of USC, Carlsbad, CA; Keck School of Medicine of USC, Staten Island, NY; Keck School of Medicine of USC, Los Angeles, CA; County of Los Angeles Fire Department, Mammoth Lakes, CA; Los Angeles County Fire Department, Monterey Park, CA; University of Southern California, Los Angeles, CA; Keck Medicine of USC, Los Angeles, CA; Keck School of Medicine of USC, Carlsbad, CA; Keck School of Medicine of USC, Staten Island, NY; Keck School of Medicine of USC, Los Angeles, CA; County of Los Angeles Fire Department, Mammoth Lakes, CA; Los Angeles County Fire Department, Monterey Park, CA; University of Southern California, Los Angeles, CA; Keck Medicine of USC, Los Angeles, CA; Keck School of Medicine of USC, Carlsbad, CA; Keck School of Medicine of USC, Staten Island, NY; Keck School of Medicine of USC, Los Angeles, CA; County of Los Angeles Fire Department, Mammoth Lakes, CA; Los Angeles County Fire Department, Monterey Park, CA; University of Southern California, Los Angeles, CA; Keck Medicine of USC, Los Angeles, CA; Keck School of Medicine of USC, Carlsbad, CA; Keck School of Medicine of USC, Staten Island, NY; Keck School of Medicine of USC, Los Angeles, CA; County of Los Angeles Fire Department, Mammoth Lakes, CA; Los Angeles County Fire Department, Monterey Park, CA; University of Southern California, Los Angeles, CA; Keck Medicine of USC, Los Angeles, CA; Keck School of Medicine of USC, Carlsbad, CA; Keck School of Medicine of USC, Staten Island, NY; Keck School of Medicine of USC, Los Angeles, CA; County of Los Angeles Fire Department, Mammoth Lakes, CA; Los Angeles County Fire Department, Monterey Park, CA; University of Southern California, Los Angeles, CA; Keck Medicine of USC, Los Angeles, CA; Keck School of Medicine of USC, Carlsbad, CA; Keck School of Medicine of USC, Staten Island, NY; Keck School of Medicine of USC, Los Angeles, CA; County of Los Angeles Fire Department, Mammoth Lakes, CA; Los Angeles County Fire Department, Monterey Park, CA; University of Southern California, Los Angeles, CA; Keck Medicine of USC, Los Angeles, CA; Keck School of Medicine of USC, Carlsbad, CA; Keck School of Medicine of USC, Staten Island, NY; Keck School of Medicine of USC, Los Angeles, CA; County of Los Angeles Fire Department, Mammoth Lakes, CA; Los Angeles County Fire Department, Monterey Park, CA; University of Southern California, Los Angeles, CA; Keck Medicine of USC, Los Angeles, CA; Keck School of Medicine of USC, Carlsbad, CA; Keck School of Medicine of USC, Staten Island, NY; Keck School of Medicine of USC, Los Angeles, CA; County of Los Angeles Fire Department, Mammoth Lakes, CA; Los Angeles County Fire Department, Monterey Park, CA; University of Southern California, Los Angeles, CA; Keck Medicine of USC, Los Angeles, CA; Keck School of Medicine of USC, Carlsbad, CA; Keck School of Medicine of USC, Staten Island, NY; Keck School of Medicine of USC, Los Angeles, CA; County of Los Angeles Fire Department, Mammoth Lakes, CA; Los Angeles County Fire Department, Monterey Park, CA; University of Southern California, Los Angeles, CA; Keck Medicine of USC, Los Angeles, CA

## Abstract

**Introduction:**

The percent total body surface area (TBSA) burned is a critical determinant of required level of care, initial management, and prognosis in burn patients. The current gold standard for TBSA estimation, the Lund-Browder (LB) chart, requires familiarity with its construction and is not necessarily feasible in the field. Recently, many mobile applications (apps) have been developed to calculate TBSA. However, few have been rigorously validated. In this study, we present a novel TBSA calculator app developed for first responders and validate its accuracy.

**Methods:**

Infant, pediatric, and adult mannequins were fabricated with eight simulated burns (Figure 1). Thirteen first- and second-year medical students with no experience in burn care were tasked with calculating the TBSA of these burns using both LB and our app. Students then completed a questionnaire to assess their experience using both methods. A paired t-test was used to compare absolute mean differences in TBSA between student estimates using both methods and those of two expert burn surgeons using LB.

**Results:**

The app performed significantly better than LB for three of the simulated burns with mean TBSA values better approximating expert values (Burn 2 p = 0.002, Burn 5 p = 0.039, and Burn 8 p = 0.006). For the remaining five simulated burns, there was no significant difference in performance between the two methods (Table 1). Students overwhelmingly reported the app was easier and faster to use (92%, n = 12), and universally preferred it over LB for the calculation of TBSA. Students cited the visual nature of the app interface and the lack of need for manual calculations as reasons for their preference.

**Conclusions:**

The app offered an easier, faster, and more accurate alternative to LB for calculation of TBSA among inexperienced users. Future directions include replicating this study with first responders.

**Applicability of Research to Practice:**

Our app may be a valid alternative to LB for TBSA calculation.